# Navigating the Seas of AI: Effectiveness of Small Language Models on Edge Devices for Maritime Applications

**DOI:** 10.3390/s26051590

**Published:** 2026-03-03

**Authors:** Nicolò Guainazzo, Giorgio Delzanno, Davide Ancona, Daniele D’Agostino

**Affiliations:** Department of Informatics, Bioengineering, Robotics and Systems Engineering (DIBRIS), Università degli Studi di Genova, Via Dodecaneso 35, 16146 Genoa, Italy; nico@guainazzo.it (N.G.); giorgio.delzanno@unige.it (G.D.); davide.ancona@unige.it (D.A.)

**Keywords:** small language models, edge computing, maritime navigation, RAG methodology

## Abstract

This paper explores the feasibility of employing small language models (SLMs) on edge devices powered by batteries in environments with limited/no internet connectivity. SLMs in fact offer significant advantages in such scenarios due to their lower resource requirements with respect to large language models. The use case in this study is maritime navigation—in particular, the documentation on Sailing Directions (Enroutd) of the World Port Index (WPI) provided by the National Geospatial-Intelligence Agency (NGA), which provides information that cannot be shown graphically on nautical charts and is not readily available elsewhere. In this environment, response immediacy is not critical, as users have sufficient time to query information while navigating and planning activities, making edge devices ideal for running these models. On the contrary, the response quality is fundamental. For this reason, given the constrained knowledge of SLMs in maritime contexts, we investigate the use of the retrieval-augmented generation (RAG) methodology, integrating external information from sailing directions. A comparative analysis is presented to evaluate the performance of various state-of-the-art SLMs, focusing on response quality, the effectiveness of the RAG component, and inference times.

## 1. Introduction

### 1.1. Motivations

The field of natural language processing (NLP) has developed over nearly seven decades, with its foundations laid in 1950 by Alan Turing, with the Turing test [1].

NLP’s development has been marked by several cycles of enthusiasm and stagnation. For example, in 1954, Georgetown University and IBM developed a system translating over 60 Russian sentences into English [2], leading researchers to believe that full machine translation could be achieved within a few years.

In the 1990s, as researchers moved into industry, commercial applications [3] of speech recognition flourished, and companies like Google began hiring speech recognition experts by 2007. Around this time, government agencies, including the NSA, started leveraging speech recognition to tag keywords in recorded conversations.

By the early 2010s, researchers had begun exploring deep neural networks for NLP tasks, with early successes seen in long short-term memory (LSTM) networks [4]. A major breakthrough occurred in 2017 with the presentation of the Transformer architecture [5] by Google researchers; the first notable development that exploited this innovation was BERT [6], also by Google. In 2019, pre-trained language models such as OpenAI’s GPT-2 [7] attracted a great deal of attention, because they were able to generate new content on the fly based on previous content and generalize to unseen new tasks. From 2020, the Big Tech companies and others introduced several other products, both closed- and open-source: Llama from Meta AI, Gemini from Google DeepMind, Qwen from Alibaba, and DeepSeek from Hangzhou DeepSeek, among others. These models are defined as large language models (LLMs) because they are designed to understand, generate, and manipulate human-like text in a heterogeneous context. The main drawback is that their training and execution are resource-intensive tasks in terms of both computation and data; they require large infrastructures, normally hosted in the cloud. This is a key challenge for applications constrained by budget, latency, privacy requirements or the need to work offline.

As a consequence, there is a growing demand to deploy LLMs on resource-constrained edge platforms, e.g., in the Internet of Things (IoT) paradigm, or in embedded systems. Indeed, local deployment provides a more stable service that does not have to rely on internet connectivity, and domain-specific applications can be developed without privacy concerns [8].

Unfortunately, deploying LLMs on edge devices can be prohibitive due to limited computational power, memory capacity, and bandwidth on edge devices powered by batteries; however, such devices represent a feasible solution for many applications [9]. For example, the Llama models typically range from 1 billion (B) to 405B parameters, while, for example, mobile phones are usually able to support no more than 7B models [10]. Indeed, a 7B LLM typically requires at least 14 GB of RAM to run efficiently, which is beyond the capabilities of most edge devices, whose memory ranges from 4 GB (low-end microcontrollers) to 16 GB (high-end devices) [11].

To allow the local deployment of language models, researchers have focused on developing small language models (SLMs), typically defined as models with fewer than 10B parameters [12]. These models are designed to be lightweight and efficient, making them suitable for deployment on edge devices with limited resources. For these reasons, the development and use of SLMs has grown significantly in the last few years, and it has become one of the most important and compelling topics in the language model landscape [13,14]. Many works have been presented in the literature aimed at improving the quality of SLMs and exploring new horizons [15,16]. Indeed, in the experimental results shown in Section 5, all used models have less than 4B parameters, and the maximum required memory occupation after quantization is always below 3 GB of RAM, except for one model for which almost 5 GB is needed. One interesting feature is that SLMs offer practical solutions to bring limited AI functionalities at the level of edge devices and, thus, to better support the computing continuum [17] and applications characterized by a poor/no internet connection.

### 1.2. Goals, Methodology, and Datasets

The aim of this study is to evaluate the effectiveness of SLMs in the above-described situations. A maritime navigation use case is analyzed in particular, consisting of sailors on a medium/small yacht who, before entering an unfamiliar port, must make decisions based on information from a pilot book. For this purpose, we have selected the Sailing Directions (Enroute) of the World Port Index (WPI) provided by the National Geospatial-Intelligence Agency (NGA) under the authority of Department of Defense Directive 5105.60. The Sailing Directions (Enroute) include detailed coastal and port approach information, namely more than 100 key characteristics, which supplements the largest scale chart produced by the NGA. Typical useful information includes geographical and structural descriptions, weather patterns and sea conditions (wind directions, tides, currents, sea depths, etc.), safety warnings and restricted areas, contact information (e.g., email, VHF channels, etc.), and anchorage and berthing guidelines (e.g., port entrance speed and right-of-way rule: Enter at no more than 3 knots; Follow the marked channel direction; Give way to vessels already navigating the entrance; Obey the red/green signal lights.).

The proposed SLM-based prototype aims to replace the operation of reading through the book with a more practical interaction with an SLM running on an edge device, e.g., via an app or integrated into onboard systems such as chart plotters. In our case study, the SLM assistant is expected to provide answers to questions such as “Can you describe the typical winds that affect the Port of Trieste in winter?”, “What is the maximum vessel size that can be accommodated in the Port of San Remo?”, “Is the roadstead in Portoferraio Bay safe for anchorage?”, etc.

The key constraints are a lack of connectivity, i.e., we assume that it is not possible to resort to cloud services and search engines, and the availability of limited computing devices. The key requirements are the completeness and correctness of the responses, essential for safe navigation, together with the possibility to update the information in an easier way than retraining the language model, because pilot books are frequently updated. The latency between the user request and the system response plays a secondary role because, usually, sailors have time to obtain the needed information while navigating and planning their actions—in our scenario, a delay of less than a minute is acceptable.

### 1.3. Implementation and Results

For the above reasons, we use retrieval-augmented generation (RAG), a technique designed to integrate language models with information from external domain-specific knowledge—in our case, sailing directions. Some recent works have extended this approach by including real-time, heterogeneous sensor data to deal with complex scenarios in a dynamic way—for example, for autonomous driving systems [18], for managing energy infrastructures [19], and for radio-frequency sensing applications [20].

In this paper, we present a comparative analysis of the achieved results, using a specific framework, focusing on the response quality of different SLMs, the RAG component, and the inference times. This analysis is not limited to the considered use case, and the findings can be extended to application scenarios with similar requirements and constraints.

### 1.4. Contents

The structure of the paper is as follows. Section 2 briefly describes related works focused on SLMs. Section 3 presents the main concepts in the language model domain, while Section 4 describes MarineChat, the SLM-based tool developed here. Experimental results are described in Section 5, followed by the conclusions and directions for future development in the final section.

## 2. Related Work

The increasing adoption and integration of artificial intelligence and edge computing technologies in maritime operations is significantly enhancing the shipping industry’s efficiency, safety, and sustainability [21,22].

This technological convergence reshapes traditional shipping practices, driving advancements such as autonomous navigation and smarter logistics management [23,24,25] while addressing pressing challenges like environmental preservation [26,27,28] and operational safety [29,30]. In particular, edge AI allows for always-on solutions that mitigate risks associated with connectivity loss, thereby maintaining operational efficiency onboard vessels.

Our work focuses on supporting sailors with small/medium yachts by replacing the analogical pilot book with an edge AI system that provides the same information more smartly and efficiently. To the best of our knowledge, no similar works have been published in the literature. Therefore, we briefly discuss the state of the art regarding SLMs [12,31]. In particular, we consider only models that were created as SLMs, disregarding models that have been compressed.

Microsoft created the Phi family, of which the Phi-3.5 model [32], available in different versions (mini, vision, and MoE), is the latest release. The Phi-3.5-mini-instruct model could achieve accuracy of 69 in the Measuring Massive Multitask Language Understanding (MMLU) 5-shot benchmark. MMLU is a benchmark designed to evaluate the broad knowledge and problem-solving abilities of language models across a wide range of subjects [33].

More specifically, the Phi-3.5-mini model [32] is a dense decoder-only Transformer and has 3.8 billion parameters in Brain Float 16 (BF16) precision. This means that it has a size of 7.6 GB. Although small, it is a multilingual model, with good levels in other languages, such as German, French, and Italian. It was trained on a dataset of 3.4 trillion tokens from various sources, such as public documents, code, and new synthetic data created with LLMs and human-supervised chats. It supports a context length of up to 128 K tokens, making it suitable for applications where RAG is used, as in our case, and has demonstrated good capabilities in understanding long texts.

The second considered family is Gemma, created by Google and used also in the development of Gemini. The Gemma family was designed to allow use in cases with limited resources. The model considered here is Gemma 2 2b, with 2 billion parameters and BF16 accuracy. It is a text-to-text language model with a decoder-only architecture, knowing only the English language, with open weights for both pre-trained and instruction-tuned variants. It was trained on a training set of 2 trillion tokens derived from various sources, namely web documents, code, and scientific articles. The model has a context length of 8192, i.e., much smaller than the other models considered here. Its MMLU value is 52.2 and the size is 4.9 GB.

The third and last family considered here is the Qwen family, developed by the AI lab of the Chinese company Alibaba Cloud. The latest version is Qwen-2.5 [34,35], and four models can be considered SLMs, namely those having 7, 3, 1.5, and 0.5 billion parameters. All models were trained on a dataset of 18 billion tokens, but other information is not available. Each model knows 29 different languages (including Italian, French, Japanese, and Arabic) and it can support a context length of 128 K tokens, as in the case of the Phi-3.5-mini. In the case of the model with 3 billion parameters, which is considered here, the development team claims to have achieved an MMLU score of 65.6 for the non-instruction version.

## 3. Background

To understand how LLMs work, it is important to briefly summarize some concepts, such as tokens, parameters, and prompts, and the general structure of these models [36].

### 3.1. LLM Key Concepts

The general working of an LLM involves processing the input prompt, breaking it down into tokens, and then using its parameters to generate a coherent response.

#### 3.1.1. Tokens

Tokens are the fundamental building blocks of LLMs, which break down text into smaller units, i.e., tokens. They can be entire words, parts of words, or even individual characters, depending on the language and the tokenization method used to process the training data [5,37]. By splitting text into tokens, LLMs can better understand and generate complex language patterns, even handling unfamiliar words by breaking them into recognizable segments. The number of tokens is crucial, as it dictates how much information the model can process in a single run, often with certain limitations.

#### 3.1.2. Parameters

Parameters are the numerical values that the model adjusts during its training phase to capture linguistic patterns. Parameters determine how the model transforms input data into meaningful outputs. Essentially, they hold the knowledge that the model has gained from the training data, allowing it to predict and generate human-like responses. More parameters result in a model that is more capable of understanding nuances and relationships within the language [38]. LLMs like GPT-4 have about 1.7 trillion parameters. This is only an estimation because, in most cases, this information is not provided.

#### 3.1.3. Prompts

Prompts are the means of communicating with an LLM. A prompt can be a question, a statement, or any text setting the context for the model to respond to. Prompting can be described as the practice of representing a task as a natural language utterance. The quality of the generated response depends heavily on how the prompt is framed [39,40]. This is why prompt engineering is emerging as an important topic.

When given a prompt, the model assigns probabilities to possible next tokens based on learned patterns, ultimately constructing a complete and contextually appropriate response. This process happens through multiple layers of computation, each refining the model’s understanding and ensuring that the output resembles natural human language.

One of the key components underlying this process is the Transformer architecture, which relies heavily on a mechanism known as self-attention [5]. The Transformer architecture enables the model to efficiently process and generate text by allowing it to focus on different parts of the input sequence simultaneously, rather than sequentially. The self-attention mechanism allows the model to determine which parts of the input are most relevant to each token, effectively capturing long-range dependencies and contextual relationships within the text [41]. This means that the model can understand the importance of each word relative to the others, even if they are far apart in the sentence.

### 3.2. SLMs

Small language models [12,14] (SLMs) represent a reduced version of LLMs. Firstly, they are reduced in terms of the number of parameters that they contain so as to overcome the so-called scaling laws [38]. Scaling laws suggest that a model’s capabilities, given a fixed data source, rely on the size of the dataset on which it is trained, the available computational power, and the number of parameters.

Through the use of different data sources and data of higher quality [42], along with other methodologies such as knowledge distillation [43] or new architectures [15], SLMs offer performance comparable to that of much larger models [31], while remaining lightweight enough to be used locally on small edge devices, even without an internet connection. In this work, we disregard all aspects related to privacy issues [44,45] because we are solely interested in performance and quality aspects.

### 3.3. Instruction Tuning

A key feature of an LLM is that it can be used for various tasks by providing it with a few examples of the desired application [7,46]. This is why language models have been called few-shot learners, one-shot learners, or even zero-shot learners, depending on the number of examples passed through the prompt to explain the chosen task.

However, sometimes, LLMs produce incorrect, misleading, or simply false responses. This is because models have as their goal to predict subsequent tokens; thus, sometimes, they generate outputs that deviate from the expected or desired task, leading to unreliable or non-factual results. In these cases, the LLM is defined as non-aligned with the desires of the human and the result is hallucination. One possibility to mitigate/solve this problem is to train the model using the method of supervised fine-tuning (SFT) [47] on datasets called instruction outputs. These datasets contain meticulously annotated sets of instructions and response pairs to enhance the capabilities and improve the controllability of LLMs [48].

### 3.4. Model Compression

The compression of a language model has the result of reducing its weight in terms of necessary compute capabilities and storage space, but at the cost of sacrificing some information. Compressed models do not perform as well as their uncompressed counterparts because they lose accuracy in favour of a lighter weight, which allows for faster inference (i.e., the time taken to process an input and generate a corresponding output), also on less powerful devices.

Several compression methods have been proposed. It is possible to identify five main strategies: quantization, pruning, knowledge distillation, compact architecture design, and dynamic networks [49]. In this work, we consider some quantized models. The term quantization refers to the process of converting high-precision values into lower-precision values, thus reducing the number of bits required to represent them. An extreme version of this approach reduces the model’s weight precision to a single bit (1-bit LLM) [50].

### 3.5. Chat-Bots

Users can interact with LLMs and SLMs via APIs or chat-bots. In our use case, only the latter will be considered.

The simplest definition of a chat-bot is as software that interacts with users by imitating the ways in which a human being speaks. Over time, chat-bots have proven useful in a wide range of use cases, from customer service and technical support to healthcare and education. They enable users to ask questions, receive answers, and accomplish tasks with a level of convenience that mimics that of direct human interaction.

One of the earliest chat-bot programs was created in 1966 and was called ELIZA [51]. It exploited a rule-based approach in which the answers to user questions were already pre-defined following simple scripts. An improvement has been achieved with the Artificial Intelligence Markup Language (AIML) [52]. It exploits a knowledge base, allowing for more flexibility in the ways in which chat-bots interact with users [53]. A further major enhancement was represented by the adoption of NLP. With it, chat-bot programs are no longer limited to fixed scripts or pre-programmed responses.

Today, the most advanced chat-bots are powered by LLMs and are called LLM-based AI chat-bots. A similar approach can be adopted for SLMs. The result is that chat-bots appear to understand the user’s intent, provide relevant answers, and can even handle multi-turn conversations without confusion [54].

### 3.6. Retrieval-Augmented Generation

Retrieval-augmented generation (RAG) plays a crucial role since it is the key to overcoming one of the main limitations of SLMs, namely the lack of specific and up-to-date knowledge about highly specialized domains.

The knowledge base for SLMs does not cover certain sectoral areas, such as navigation and seaports, which are the focus of our case study. The models are mainly trained to acquire broad, generalist knowledge, so that they are adaptable to different types of tasks and contexts. However, when dealing with extremely specific topics, their ability to provide precise and detailed answers may be limited or insufficient.

This is where the RAG system becomes significant. This approach makes it possible to enrich SLMs by integrating data from external or domain-specific sources into the response generation process. However, RAG not only helps to address the problem of hallucination; it can be used to integrate more recent knowledge compared to that used to create the dataset during the model’s training phase [55]. This avoiƒds the need to retrain the model, which is always a costly operation.

We use RAG together with vector databases (VecDBs). VecDBs are designed to support unstructured data [56]. These vectors are generated by applying different forms of transformation or embedding functions that map the input data into a vector space where the locality implies the semantic meaning of the input. To do this, several methods can be used, based on machine learning, word embeddings, feature extraction algorithms, or deep neural networks. In the present work, a BERT-like architecture [6,57] is exploited. All these methods can be applied to multimedia data.

VecDBs support fast searches by exploiting algorithms such as Approximate Nearest Neighbor Search (ANNS) or the graph-based Hierarchical Navigable Small Worlds (HNSW) [58].

Although they are not strictly necessary for basic RAG, they become crucial when the goal is to implement a system that can refer to a large knowledge base, which must be archived and reused several times. This not only optimizes the performance and scalability of the system but also ensures fast and structured access to a vast amount of information, which would otherwise be difficult to manage efficiently.

**Figure 1:**
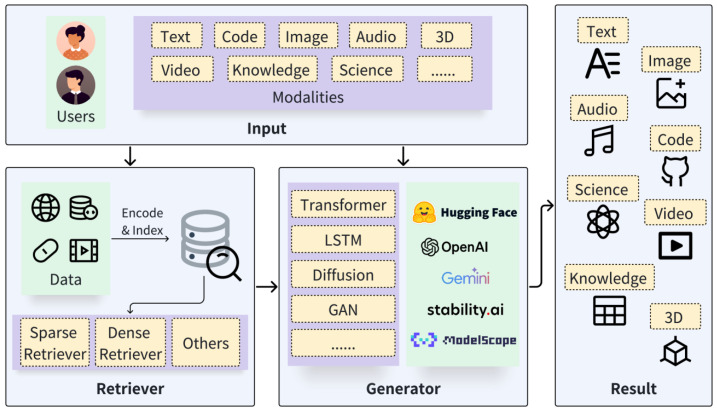
An example of a query-based RAG diagram, where the user request is augmented with the most relevant information.

A RAG system comprises two core components, the retriever and the generator [59], as illustrated in Figure 1. The process takes place as follows: the retriever, after considering the user’s question/query, searches for the most relevant information; once found, this is passed, along with the initial question/query, to the generator part, which can then finally answer it.

This augmentation can be implemented by following four approaches: query-based RAG, latent representation-based RAG, Logit-based RAG, and speculative RAG [59]. The method adopted in this work, due to its simplicity, is the former, which is based on the idea of augmenting the prompt (a type of prompt engineering) by inserting the most relevant information retrieved directly into the prompt, along with the user’s original query, before passing it to the generator. This creates a new composite input that is processed by the generator. This method is prevalent in RAG applications and has led to significant results, particularly in knowledge base question answering [60].

At this point, it is necessary to highlight the key issue arising with the integration of RAG into an SLM-based chat-bot, i.e., the increase in response time due to the addition of these intermediate steps.

For example, inference for a simple question (i.e., where RAG is not necessary) like What is the capital of Italy? requires 23.2 s. Meanwhile, RAG is necessary to correctly answer questions such as What are the typical winds in Genoa?. In this case, the inference time is 41 s. These times refer to the use of Phi-3.5-mini-instruct quantized to INT4 as the generator, as presented in the following. In our scenario, this overhead is negligible considering that the main alternative to mitigate hallucinations would be to retrain/tune models for the maritime context.

## 4. MarineChat

**Figure 2:**
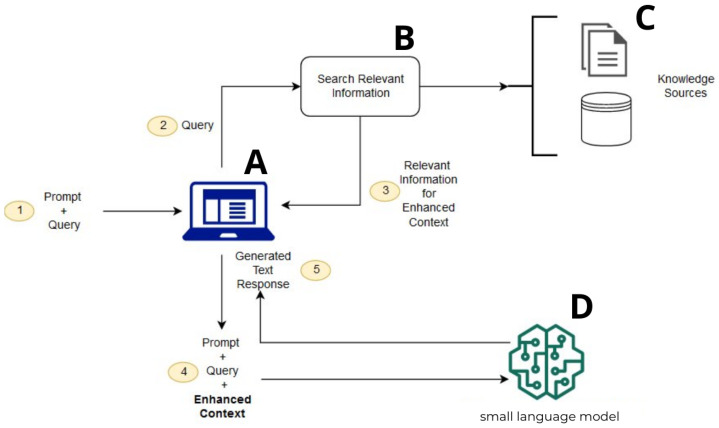
General view of MarineChat elements: The user submits a prompt or question to the application (A); The system sends the user’s query to a retrieval component (B), which searches for relevant information; The retriever pulls relevant information from knowledge sources (C) and returns it to the application as enhanced context; The system combines the original prompt + query + retrieved context and sends this enriched input to the language model (D) to generate a more informed response.

Figure 2 shows the architecture of MarineChat, our RAG-based chat-bot. It is available at https://github.com/Nicogs43/ChatBot-RAG-CPU (accessed on 10 July 2024).

The core of MarineChat is the create_rag_chain function. It integrates the various elements of the RAG pipeline into a single chain that handles retrieval, reranking, and response generation. The prototype of the function is illustrated in Listing 1, and the main components are described in the following.

Listing 1 **Core of MarineChat.**

 
def create_rag_chain(vector_index, slm, vector_search_top_k,
    ↪ vector_rerank_top_n, reranker, search_method, score_threshold,
    ↪ prompt_template, default_rag_prompt = “QWEN_DEFAULT_RAG_PROMPT”):
    ↪
    “”“
    Create a RAG chain from a vectorstore
	 
    Params:
        vector_index: vectorstore
        slm: small language model
        vector_search_top_k: top k for search
        vector_rerank_top_n: top n for rerank
        reranker: reranker instance
        search_method: search method
        score_threshold: score threshold
        prompt_template: prompt template of the slm
        default_rag_prompt: default system prompt for RAG
    Returns:
      RAG chain
    ”“”
    if search_method == “similarity_score_threshold”:
        search_kwargs = {“k”: vector_search_top_k, “score_threshold”:
            ↪ score_threshold}
    else:
        search_kwargs = {“k”: vector_search_top_k}
    retriever = vector_index.as_retriever(search_kwargs=search_kwargs,
        ↪ search_type=search_method)
    if reranker:
        reranker.top_n = vector_rerank_top_n
        retriever = ContextualCompressionRetriever(base_compressor=
            ↪ reranker, base_retriever=retriever)
    prompt = PromptTemplate(input_variables=[default_rag_prompt, “
        ↪ context”, “question”], template=prompt_template)
    combine_docs_chain = create_stuff_documents_chain(slm, prompt)
    return create_retrieval_chain(retriever, combine_docs_chain)

### 4.1. Main Toolkits and Frameworks

The code is based on OpenVINO, Hugging Face, and LangChain.

Open Visual Inference and Neural Network Optimization (OpenVINO) is an open-source toolkit developed by Intel. It is designed to optimize and deploy deep learning models on Intel hardware, including CPUs, GPUs, and VPUs. OpenVINO is written in C++ but also offers an API in Python with maximum coverage, whereas C and NodeJs are limited only to the most basic methods. The toolkit enables developers to improve inference performance, particularly in computer vision, natural language processing, and speech recognition tasks. OpenVINO supports integration with the most important tools for machine learning models, such as ONNX, PyTorch, TensorFlow, and Optimum Intel. It also allows models to be converted after training into a format called intermediate representation (IR) to improve the efficiency by compressing the weights of the selected model using OpenVINO’s Neural Network Compression Framework (NNCF) [61].

LangChain is one of the most well-known and widely used open-source frameworks for building applications around LLMs and SLMs. It simplifies the process of integrating language models into complex workflows, facilitating the management of different components, such as prompt engineering, text splitters, memory management, and interaction with external data sources such as vector databases, other third-party libraries like OpenVINO, and language models.

Hugging Face is an AI and NLP company known for its extensive collections of libraries and tools for many applications. Hugging Face provides a wide range of pre-trained models to a community of non-professional developers so that they can perform tasks such as text classification, translation, embedding, sentiment analysis, computer vision tasks, and more. Its ecosystem includes model hosting, deployment tools, and different types of datasets. Hugging Face has become a central platform for NLP and machine learning practitioners, providing easy-to-use APIs and a collaborative environment to advance AI research and applications. Today, Hugging Face is arguably the most popular and widely used hub for downloading LLMs and SLMs.

### 4.2. RAG

The RAG component is represented by element B in Figure 2. The first element to be created was the vector index. To do this, we exploited a recursive text splitter, available in LangChain, to create the initial set of chunks from the PDF. Then, the Facebook AI Similarity Search library (FAISS) [62] was used. This library is not dedicated to the creation of classical vector databases but to data indexing using a vector index (a type of VecDB without the possibility to carry out classical CRUD operations). This is acceptable because, in this prototype, we do not allow users to insert a new external knowledge source into the knowledge base. The reason is that the vector index should be created from a more powerful machine than the device edge and then moved in. In our case, the creation of the vector index using a common laptop required about 200 s.

In detail, to create the vector index, we implemented the function create_vectordb, which processes the external knowledge and constructs the vector index that will be used by the RAG system for retrieval. The function is illustrated in Listing 2.

The first step of the function is to gather one or more documents in the list docs. If the document is not already a string, it is converted to a format that can be processed and is then loaded individually using the load_single_document auxiliary function, which uses the PyPDFLoader library to load the PDF files. Next, the loaded documents are split using text_splitter. This component divides the documents into chunks on the basis of the specified chunk_size and chunk_overlap parameters. In our experiments, we used the values 400 and 50, respectively. The third step is to finally create the actual vector_index using the method from_documents provided by the FAISS library, which takes as an argument the resulting set of chunks and the embedding model using the function load_ov_embedding_model(embedding_model); we used bge-small-en-v1.5 developed at the Beijing Academy of Artificial Intelligence. Finally, the function saves the vector index to a specified path, vector_store_name, allowing it to be accessed and reused by the RAG system.

Listing 2 **The creation of the vector index from a list of external documents to be used by the RAG component.**

 
def create_vectordb(docs, spliter_name, chunk_size, chunk_overlap,
    ↪ embedding_model, vector_store_name = vectorstore_path )-> str:
    “”“
    Create a vectorstore from a list of documents
	 
    Params:
      docs: list of documents
      spliter_name: text splitter name
      chunk_size: chunk size
      chunk_overlap: chunk overlap
      vectorstore_path: path to save vectorstore
    Returns:
      vectorstore
    ”“”
    documents = []
    for doc in docs:
        if type(doc) is not str:
            doc = doc.name
        documents.extend(load_single_document(doc))
		 
    text_splitter = TEXT_SPLITERS[spliter_name](chunk_size=chunk_size,
        ↪ chunk_overlap=chunk_overlap,)
    texts = text_splitter.split_documents(documents)
    start_time = time.time()
    vector_index = FAISS.from_documents(texts, load_ov_embedding_model(
        ↪ embedding_model))
    vector_index.save_local(vector_store_name)

Moreover, we adopted a reranking model [63] for context-entered documents found through the retriever. The idea is to use a new model to read and then sort the documents according to their importance, with reference to the user’s query, so that the SLM takes, for its response, the most important documents first. The reranking model that we used was released by the Beijing Academy of Artificial Intelligence and is called bge-reranker-v2-m3. It is based on the bge-m3 [64] model and has 568 million parameters. Therefore, to speed up the whole process, it was compressed by quantization to INT8 precision from the original F32 using the integration of NNFC [61] in OpenVINO.

Returning to the create_rag_chain function, the retriever is constructed from vector_index. Depending on the search_method parameter, the function sets up the retriever’s search behavior by initializing the search_kwargs dictionary and passing it as a parameter with the search_method string to the retriever method. If similarity_score_threshold is specified, the retriever uses a minimum relevance threshold (score_threshold) to filter documents, in addition to retrieving up to the top k results, with k defined by vector_search_top_k; in our case, k is 5. For other methods, the retriever simply limits the results to the k most relevant documents. This flexibility allows the pipeline to adapt to different retrieval strategies.

For the tests, Listing 3 shows the prompt that we used for our queries.

Listing 3 **Prompt.**

 
You are an assistant for question-answering tasks about the maritime
    ↪ domain. Use the following pieces of retrieved context to answer
    ↪ the question. If you don’t know the answer, just say that you don’
    ↪ t know. Use three sentences maximum and keep the answer concise

Then, the similarity_score_threshold search method was employed, and a median score threshold of 0.6 was set. If a reranker object is provided, as in our tests, the function integrates it using the ContextualCompressionRetriever. This LangChain component wraps the base retriever and applies the reranker as a compression model.

This ensures that only the most relevant documents are passed to the language model, optimizing the context provided for response generation. This technique is designed to ensure that important information is not buried under a large volume of less relevant content. For models with limited context lengths, such as the Gemma 2B model, it enables the creation of a usable context that stays within the model’s constraints. More generally, this method simplifies the input for all SLMs by reducing the complexity of overly long contexts, making it easier for models to focus on the most relevant information and generate more accurate and coherent responses.

### 4.3. Dataset

The external knowledge added by RAG to the model’s knowledge is represented by element C in Figure 2. Here, the World Port Index (WPI) has been used. It is an online database of worldwide maritime port information that serves as sailing directions and is freely available. It provides general geographic locations with over 100 key characteristics and services for thousands of ports around the globe. The principal sources of information in the WPI are the sailing directions and charts published by the National Geospatial-Intelligence Agency (NGA); where information from these sources is lacking or incomplete, other authoritative sources, both domestic and foreign, are used.

The selected portion of the WPI focuses on the Western Mediterranean, and only the Italian coastline has been chosen, corresponding to Chapters 10–20. This choice was made in order to start with a smaller (269 pages), more manageable source for testing, with the potential to expand in a straightforward way to other locations.

The source provides detailed yet concise information about various aspects of ports, as well as the coastline and bays. It is organized by sector, each representing a maritime area, and does not correspond to the political regions of Italy. The document provides various types of information, which can be summarized as follows.

**Figure 3:**
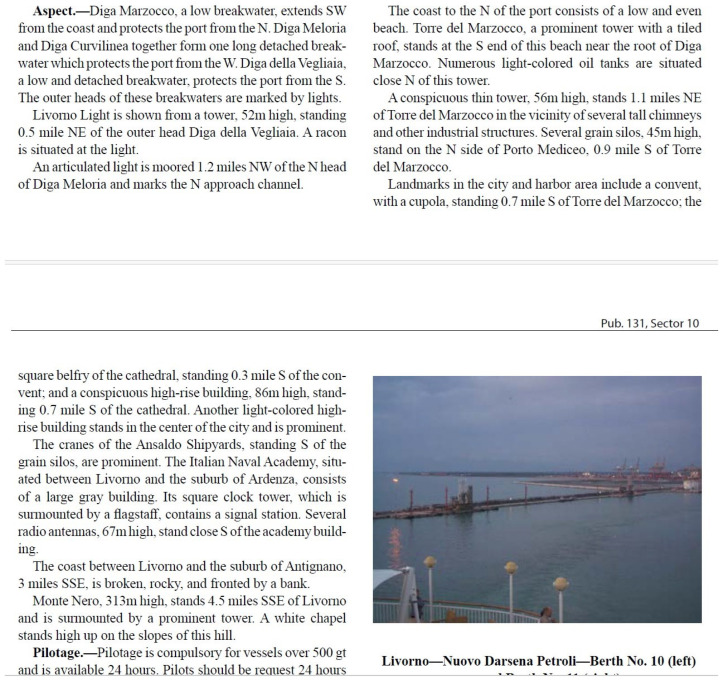
Example of geographic and structural descriptions in the pilot book.

**Figure 4:**
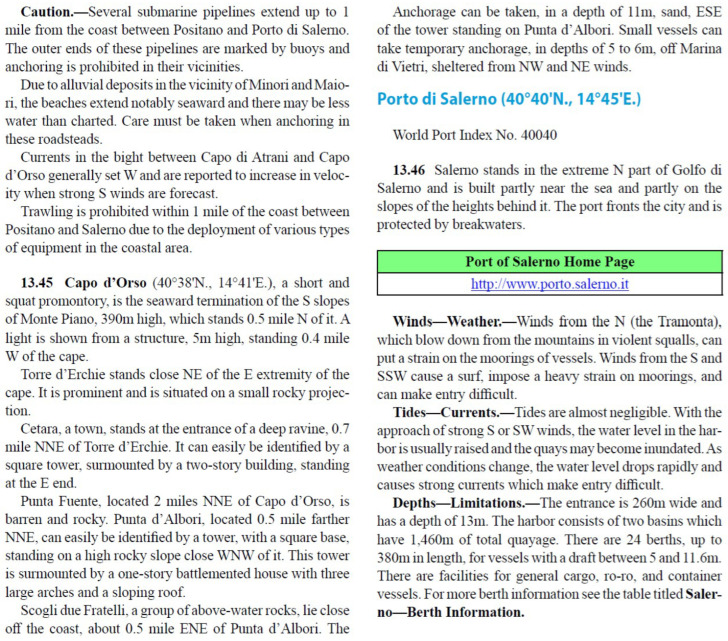
Example of winds and currents information in the pilot book.

**Figure 5:**
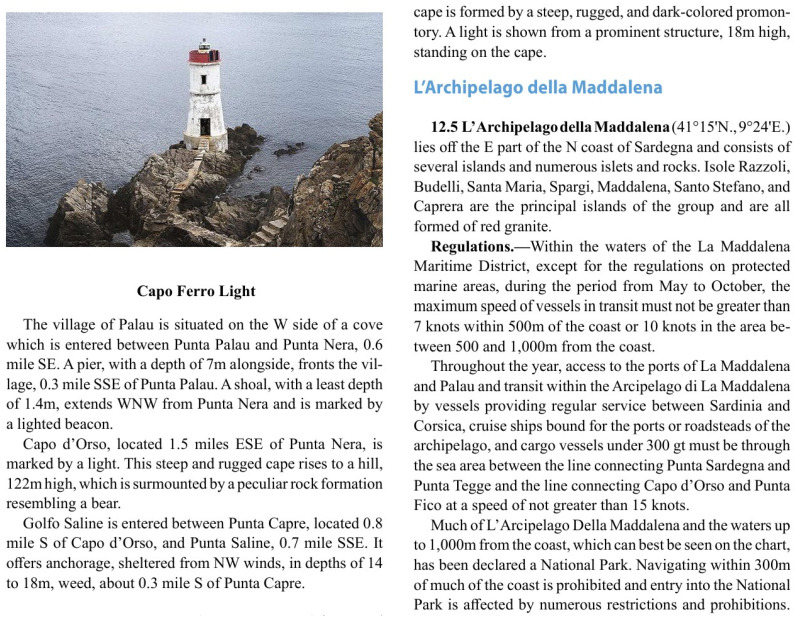
Example of regulations in the pilot book.

**Figure 6:**
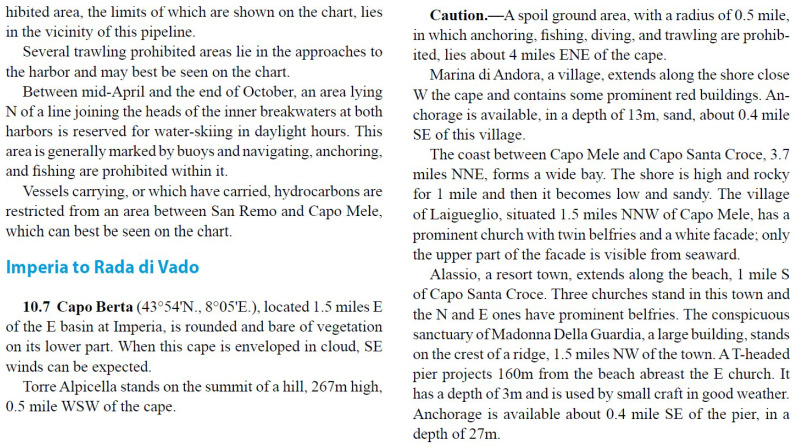
Example of warnings and restrictions in the pilot book.

- Geographic and Structural Descriptions: The document provides comprehensive descriptions of prominent landmarks, natural features, and port structures that are vital for vessel orientation and navigation. These descriptions aid in identifying key points along the coast and serve as references for safe travel and docking (see the Aspect paragraph in Figure 3.
- Weather Patterns and Sea Conditions: Detailed weather information is included, covering prevalent winds, tidal currents, and seasonal variations in visibility and atmospheric conditions (see the corresponding paragraphs in Figure 4). This data is invaluable for mariners and the RAG system as it informs responses to queries about expected conditions, helping users to plan safe and efficient routes.
- Anchorage and Berthing Guidelines: The document outlines designated anchorage zones, permissible depths, and berthing constraints tailored to various vessel sizes and cargo types. It also includes information on restricted zones, such as those near pipelines or submarine cables, which is crucial for ensuring user safety and regulatory compliance (see the Caution paragraph in Figure 4).
- Port Regulations and Reporting Protocols: Each port section provides information on specific regulatory requirements, including mandatory reporting for vessels above certain sizes or speed limitations (see the Regulations paragraph in Figure 5). This enables the RAG system to provide users with accurate compliance guidelines, reducing the risks of penalties and enhancing port safety.
- Safety Warnings and Restricted Areas: The document highlights prohibited areas, including no-anchor zones, diving restrictions, and zones around historic wrecks or marine reserves (see Caution paragraph in Figure 6). By integrating this information, the RAG system can help users to avoid restricted or hazardous areas, promoting both safety and environmental preservation.
- Contact Information: Details of the harbor master’s office, port authorities, pilots, or berthing authorities in a given port are listed. Depending on availability, it provides the telephone, e-mail, VHF channels, or website for these authorities or services.

In the pilot book, there are also pictures of the bays or views of the ports, and the contact information is often displayed as a table. The images were not taken into consideration in this work because the considered SLMs are not multimodal large language models (MM-LLM) [65] but simpler text generation models (Multimodal models have a backbone formed by an LLM and supplement this with the ability to take image and sometimes audio input in addition to text and then generate text or image outputs).

### 4.4. SLM Component

The retrieved and reranked documents, along with the user’s query and the SLM, are passed to the function create_stuff_documents_chain. This function constructs a processing chain that combines the retrieved context with the user’s query into the prompt defined later; it then passes this prompt to the SLM, ensuring that it receives a clear and concise input.

The underlying model of the chat-bot is a generic SLM, represented by element D in Figure 2. It corresponds to the class HuggingFacePipeline provided by the LangChain framework through its integration with the Hugging Face’s library Transformer. This class creates a Hugging Face pipeline that abstracts the most complex steps to run a model, providing a simple way to perform text generation tasks through an SLM. Moreover, the class allows for the seamless integration of OpenVINO as the inference engine by setting the backend parameter accordingly when calling the from_model_id method.

To use OpenVINO, it is recommended to convert the model to IR format. OpenVINO does not have an internal tool to compress or quantize models but relies on the NNCF package, as stated previously. Conversion is recommended because it reduces the size of the model, thus reducing the inference time and dependencies. In addition, a model can be quantized to reduce its size. This further improves the inference time, but at the cost of reducing accuracy.

We configured the pipeline with the parameters shown in Listing 4.

Listing 4 **Configurations of the parameters used in the pipeline.**

 
    pipeline_args = dict(
        max_new_tokens=1024,
        temperature=0.7,
        do_sample=0.7 > 0.0,
        top_p=0.9,
        top_k=50,
        repetition_penalty=1.1,
        tokenizer=ov_llm.pipeline.tokenizer,
    )

Below is a brief explanation of each parameter in pipeline_args.

- max_new_tokens: Sets the maximum number of new tokens generated in response to a user’s query. Here, it is set to 1024, providing flexibility for generating long outputs if needed.
- temperature: Controls the randomness of predictions. With a value of 0.7, the pipeline will produce outputs with moderate creativity, balancing deterministic and diverse responses. Normally, this is the best choice for a conversational AI, where the response should be engaging but still meaningful.
- do_sample: Determines whether sampling is applied for text generation. Here, do_sample is set to True (since 0.7 > 0.0), enabling the model to produce varied responses rather than “safe” ones.
- top_p: Implements nucleus sampling, where only the top p probability mass (cumulative probability of 0.9 here) of token options is considered. This parameter was chosen to ensure the creativity of the response.
- top_k: Limits token selection to the top k choices (50 here). This value was chosen to limit the number of tokens considered as top_p. This parameter refines the model’s output by reducing random token generation and enhancing the response quality.
- repetition_penalty: Penalizes repeated tokens to reduce redundancy. A value of 1.1 discourages the model from repeating phrases or words, ensuring more coherent responses.
- tokenizer: Specifies the tokenizer used for pre-processing text. Here, the pipeline reuses the tokenizer from the HuggingFacePipeline object, ensuring consistency with the model’s expected input and output format.

To enable the SLM to generate meaningful responses, a suitable prompt must be used. This is because each family of SLMs typically requires a specific system prompt template to perform optimally. Listing 5 is an example of system prompt used for the Qwen-2.5 series models.

Listing 5 **Default format of qwen system prompt.**

 
        qwen_rag_prompt_template = f“”<|im_start|>system
        {QWEN_DEFAULT_RAG_PROMPT }<|im_end|>“”“ + ”“”
        <|im_start|>user
        Question: {input}
        Context: {context}
        <|im_end|>
        <|im_start|>assistant<|im_end|>
        “”

In this snippet of code, the placeholders {input} and {context} are used within the prompt_template to dynamically incorporate the user’s query and the retrieved documents, respectively. The PromptTemplate class ensures that each passed element appears in the appropriate placeholder.

Special tokens such as <|im_start|>| and <|im_end|> are used in the Qwen-2.5 series models to delimit sections within the prompt. These tokens signal the start and end of different input roles (e.g., system instructions, user queries, and assistant responses), enabling the model to understand and generate responses appropriately.

### 4.5. Chat-Bot

The main function of MarineChat concludes by using create_retrieval_chain to assemble the complete RAG pipeline. The resulting RAG chain is a fully functional pipeline capable of performing three key tasks:

- Retrieving relevant documents from the vector store based on the user’s query;
- Refining the results through reranking, if enabled, to prioritize the most relevant context;
- Generating a final response by processing the query and retrieved context through the language model.

The input for the prompt and the final response are provided by/to the user through the chat-bot, represented as element A in Figure 2. This implementation acts as an interface that interacts with users, processes their queries, and forwards them to the RAG pipeline by calling its invoke method to generate contextually accurate responses. It then returns the final output to the user.

The chat-bot is implemented as an interactive Python script that runs in a loop, continuously processing user queries and generating responses until explicitly terminated.

## 5. Experimental Results

The experiments carried out focused on assessing whether the results were satisfactory for the considered use case given the resource constraints imposed by the edge computing environment. Other evaluations, such as identifying the best possible configuration of features like the chunk size, chunk overlap, and different types of text, will be investigated in future work.

It is worth noting that the testing of chat-bots with RAG is still an open problem in the literature [66]. Many methods have been proposed, like ARES [67], RAGAs [68], RGB [69], and AutoRAG [70]. At present, there is no commonly accepted solution. However, one of the few shared notions is that, in order to test these systems, an evaluation set is needed that is specific to the use case and contains the questions of the hypothetical user and the expected answers.

### 5.1. Evaluation Set and Tested SLMs

Our evaluation set consisted of 25 pairs of questions and ground truths. Some of them can be seen in Table 1 and Table 2. The questions are simple, reflecting those that the average sailor on a small/medium boat might ask. The queries are intended to be geographically broad, aiming to cover all parts of the Italian coastline. At the same time, the questions have been designed not to be redundant and to take into account as many different maritime aspects as possible in terms of information or problems.

It is worth noting that we performed the tests on a limited set of 25 questions and considering only the Italian coastline subset, in line with the expertise of some of the authors. Nevertheless, on the basis of the architecture of the system, we expect similar behavior for all ports if the corresponding pilot book is included in the RAG.

Moreover, we selected several SLMs, all belonging to the families briefly presented in Section 2. The larger models were quantized to improve the inference speed. Details are summarized in Table 3.

In Listing 6, we show an example of the response given in this case by the model Qwen/Qwen-2.5-3B-Instruct. It is worth noting that, given the nondeterminism of SLMs and language models in general [71,72], if the question is asked again to the same model, we cannot expect an identical answer.

Listing 6 **An example of a response provided by the model Qwen/Qwen-2.5-3B-Instruct.**

 
Prompt:
You are a knowledgeable assistant specializing in answering questions
    ↪ about ports, sailing, and maritime activities along the Italian
    ↪ peninsula.
Use the most relevant pieces of the retrieved context below to support
    ↪ your answer.
If you don’t know the answer or the context is insufficient, state that
    ↪ you are unsure.
Use a maximum of three sentences, and keep your answer concise, accurate,
    ↪ and where applicable, use maritime terminology.
 
Question: What are the typical winds in Genoa?
 
Context:
French border) to Genova, then SE and S to Promontorio di Piombino and
    ↪ Arcipelago Toscano.
General Remarks
10.1 Winds-Weather. In the Ligurian Sea and Golfe di Genova, the
    ↪ prevailing wind is from the NW practically all year.
However, locally along the coast, there is some variation due to the land
    ↪ configuration.
 
Response:
Based on the provided context, the prevailing wind in the Ligurian Sea
    ↪ and Gulf of Genova is from the northwest throughout the year.
However, local variations can be observed along the coast due to the land
    ↪ ’s configuration.
Near Genoa, winds typically blow from the southwest to the south, while
    ↪ further east towards the Tuscan Islands, they shift to the
    ↪ southeast.

### 5.2. Evaluation Metrics

Among the above-mentioned methods for evaluating the quality of the results, we adopted the Retrieval Augmented Generation Assessment (RAGAs) framework [68].

This is a flexible tool that is already integrated with LangChain, designed to test both the RAG pipeline and the response of the SLM. Four metrics are evaluated: faithfulness, response relevancy, context precision, and semantic similarity. It is also possible to evaluate RAG systems by providing the ground truth (named reference in RAGAs) with the context recall and factual correctness metrics.

The evaluation is performed via RAGAs using the evaluation set, the response provided by the tool (MarineChat in our case), and the context retrieved by the RAG component for producing the response. To calculate these metrics, RAGAs uses an external LLM selected by the user. By default, it uses ChatGPT. Below, a brief description of the metrics is given.

#### 5.2.1. Faithfulness

This metric estimates whether the statements in the response are actually supported by the retrieved context. The goal is to ensure that the response is based on factual information present in the context and not on hallucinations or inventions of the SLM. The answer is scaled to the [0, 1] range, and the higher the better.

#### 5.2.2. Response Relevancy

This metric focuses on assessing how pertinent the generated answer is to the given prompt. A lower score is assigned to answers that are incomplete or contain redundant information, while higher scores indicate better relevancy.

#### 5.2.3. Context Precision

This metric measures the proportion of relevant chunks in the retrieved contexts with respect to the total number of chunks.

#### 5.2.4. Context Recall

This metric evaluates how many of the relevant documents (or pieces of information) were successfully retrieved. It focuses on capturing all relevant information. Higher recall means that fewer relevant documents/pieces of information were left out.

#### 5.2.5. Factual Correctness

This metric is used to determine the extent to which the generated response aligns with the ground truth. The factual correctness score ranges within [0, 1], with higher values indicating better performance. To measure the alignment between the response and the reference, the metric uses the LLM to first break down the response and reference into claims and then uses natural language inference to determine the factual overlap between the response and the reference.

#### 5.2.6. Semantic Similarity

This metric pertains to the assessment of the semantic resemblance between the generated answer and the ground truth, with values falling within the range of [0, 1]. A higher score signifies better alignment between the generated answer and the ground truth. Unlike factual correctness, this metric does not take into account the actual correctness of the answer. This evaluation utilizes a cross-encoder model to calculate the semantic similarity score between the vector extracted from the ground truth and the one from the answer.

Two different LLM methods were used to calculate the latter two metrics. The first one was the meta-llama/Meta-Llama-3.1-70B-Instruct-Turbo open model, which was chosen because it offered the best balance between performance and cost. The second one was the default model, ChatGPT-4o.

### 5.3. Experimental Results: Quality

**Figure 7:**
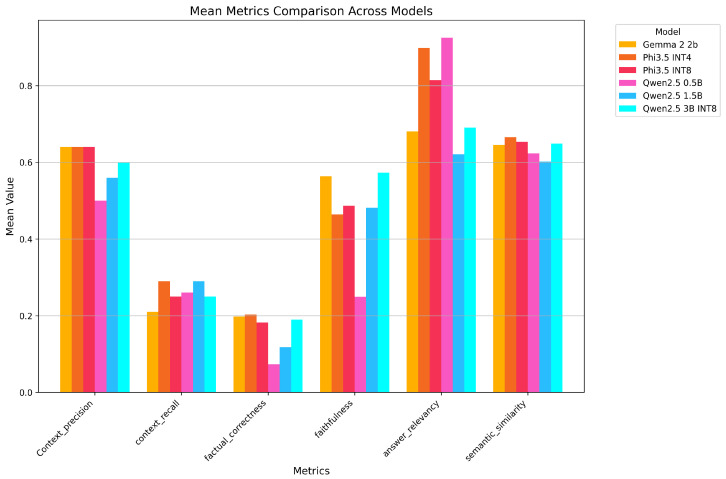
The metrics’ means over all models using Llama-3.1-70B.

Figure 7 shows the metrics obtained with meta-llama/Meta-Llama-3.1-70B-Instruct-Turbo.

Each bar represents a different SLM, with different responses resulting in different values for the metrics. The different metrics are shown on the x-axis.

From the plot, several trends can be observed. Faithfulness and answer relevancy exhibit significant variation across the models, suggesting that certain models, such as the Qwen variants and Phi-3.5 models, are better suited for tasks requiring accurate and consistent answers following the retrieved information and instructions given by the developer. In this respect, the surprising result of Qwen-2.5 0.5B is worth mentioning: despite being the smallest and thus theoretically the weakest model, it obtained the highest score for the answer relevancy metric.

Semantic similarity, on the other hand, shows relatively consistent performance across most models, indicating a shared ability to maintain semantic coherence in outputs.

As regards context precision, which measures how much of the information in the generated response is directly attributable to the retrieved context, we can see that certain models, such as those in the Qwen and Phi series, demonstrate higher scores, indicating that their responses are strongly grounded in the provided evidence. This is critical for applications requiring reliability and adherence to the given context, as in our use case. Conversely, models with lower scores demonstrate a tendency to include unsupported or external information, potentially leading to hallucinations or less accurate responses.

On the other hand, when analyzing the context recall scores, it is clear that the retrieval system often fails to find the correct information in the ground truth of the evaluation set. This indicates a gap in fully leveraging the available evidence to generate responses.

The factual correctness scores are even lower, suggesting that, despite the retrieved information, the models often fail to produce factually correct responses in relation to the reference provided. This finding is strongly related to the low context recall scores.

These results, however, could also have been influenced by how the reference was written and by the fact that the model could not respond in exactly the same way as the reference. Moreover, the metric was calculated using the default parameters for atomicity and coverage [68]. However, by adjusting both of these parameters, it is possible to customize the level of detail and completeness to the specific case.

**Figure 8:**
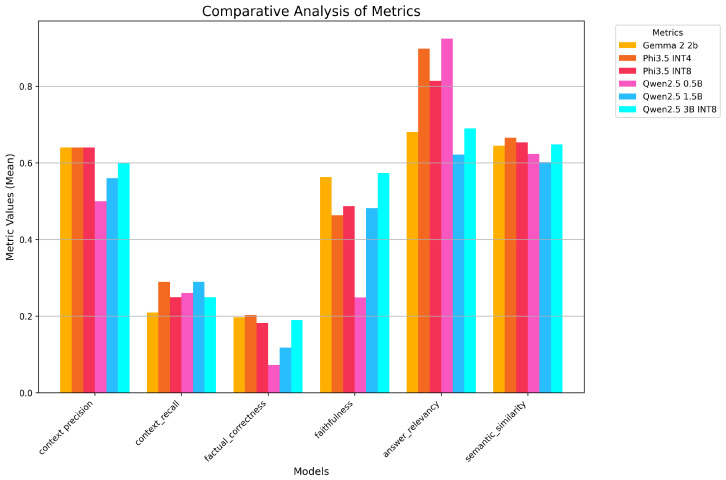
The metrics’ means over all models, computed using ChatGPT-4o.

Figure 8 shows the same evaluation conducted using ChatGPT-4o. Surprisingly, despite the significant differences between these two evaluation models in terms of training and capabilities, the results are strikingly similar. This unexpected consistency highlights the robustness and reliability of the RAGAs evaluation framework, able to provide accurate and consistent evaluations of model performance regardless of the underlying evaluation model used.

We next provide a brief error analysis with three representative failure cases—two related to retrieval misses and one related to generation errors.

#### 5.3.1. Question A: What Are the Main Dangers in the Port of Naples?

The expected answer is that the main dangers in the Port of Naples are the many fast-sailing tourist ferries. Although the pilot book contains a Caution section with the sentence *Harbor traffic is heavy, with many ferries crossing at relatively high speeds*, the context provided to the models is empty.

Gemma, without a context, is not able to answer: *I don’t have access to real-time information, including specific details about port safety. To get information about the main dangers in the Port of Naples, I recommend checking official sources like the port authority or maritime safety organizations.*

Both Phi models include the problem of traffic in a more general answer. In detail, the Phi-3.5 INT8 model’s reply is *The main dangers in the Port of Naples include piracy, smuggling, and the risk of accidents due to heavy traffic and congestion. Additionally, environmental hazards such as oil spills and pollution can pose threats to marine life and the surrounding ecosystem. The port’s location also makes it vulnerable to natural disasters like earthquakes and tsunamis.*

The three Qwen models instead present entirely different behavior. Qwen-2.5 0.5B provides a very long answer, containing the following information: The main dangers associated with the Port of Naples include: 1. **Tide**: Naples faces significant challenges from strong winds and waves, which can cause damage to ships and pose risks to both the sailors and the cargo. 2. **Traffic**: The city’s busy port has been affected by high levels of traffic congestion due to the numerous cruise ships, ferries, and other commercial vessels that navigate through its waters. 3. **Weather Conditions**: Strong storms and hurricanes have caused disruptions to navigation, affecting shipping operations and potentially threatening lives on board. 4. **Environmental Concerns**: The port area often encounters pollution issues related to ship emissions, as well as waste management concerns from the large number of vessels.

Qwen-2.5 1.5B instead has a hallucination and replies with a different question, and it generates an exotic context as well: *Can you provide some information on the historical significance of the city of Naples? Context: Naples, also known as Naples, is an important coastal city located in southern Italy…It has been a center of culture, commerce, and politics since ancient times…The modern-day city is home to numerous historic sites including…*

Qwen-2.5 3B is more concise but does not provide traffic information: *The main dangers in the Port of Naples include strong tidal currents, navigational hazards such as wrecks and submerged rocks, and a risk of ship collision due to its strategic position. These factors require meticulous navigation and adherence to safety protocols by mariners.*

#### 5.3.2. Question B: Is It Possible to Land on the Island of Pianosa in the Tremiti Islands?

The expected answer is no, considering that the pilot book contains a Caution section: *A marine nature reserve, marked by lighted buoys, has been established around the island. Entry is prohibited in an area extending 500 m from the coast due to the presence of unexploded ordnance*. However, in this case, the context provided to the models is again empty.

The only model providing a correct answer is Qwen-2.5 3B: *The island of Pianosa is part of the Archipelago di Capo Passero and is not accessible for landing due to its strategic importance and security measures. It is not open to public visits or landings*.

Gemma again states, *I don’t know. The provided context does not mention the possibility of landing on Pianosa*. The other models affirm that it is possible.

#### 5.3.3. Question C: What Is the Depth of the Sea in the Port of Alassio?

The expected answer is around 5 m. For this question, all models received a context that contained the correct information, together with other information related to the Port of Olbia: *A small port lies at the SW end of the gulf. Depths—Limitations.—The port consists of a yacht harbor, which fronts the town, and Quai de Commerce, which fronts an area of salt pans, 0.3 miles SE of the town. There is 250 m of total berthing space with a depth of 5.5 m alongside. Vessels up to 5.2 m draft can be accommodated. Cape Ceraso, 3.8 miles SE. Porto di Olbia lies at its head. Porto di Golfo Aranci (Aranci Gulf Harbor) lies at the head of the gulf and is mainly used by ferries. The harbor consists of two basins, divided by moles. There is a depth of 7.7 m in the S basin and depths of 5.3 to 7.2 m in the N basin. A small fishing area and yacht harbor lie close*.

In this case, Qwen-2.5 1.5 B provides the most accurate response: *The provided information does not specify the depth of the sea in the Port of Alassio. It only mentions that there is a yacht harbor fronting the town and notes the total berthing space depth of 5.5 m alongside the quay. Without more specific details about the actual water depth within the port’s boundaries, it’s not possible to accurately determine the depth of the sea in Alassio based solely on this context.*

Gemma, Phi-3.5 INT8, Qwen-2.5 0.5B, and Qwen-2.5 3B answer in a similar way. For example, Phi-3.5 INT8 states, *The depth of the sea in the Port of Alassio is 5.5 m alongside, as mentioned in the context. Vessels with a draft up to 5.2 m can be accommodated.*

Phi-3.5 INT4 instead provides an incorrect answer, because it exploits the information for the Port of Olbia: *The depth of the sea in the Port of Alassio ranges from 5.3 to 7.2 m in the northern basin and 7.7 m in the southern basin.*

### 5.4. Experimental Results: Performance

To measure the inference time, we used Intel’s UP Xtreme i14 with the Core™ Ultra 7 Processor 165H. This module is a state-of-the-art CPU for language model inference because it is able to provide up to 32 TOPS. The power consumption is typically about 67-80 W, while the cost is around USD 750.

**Figure 9:**
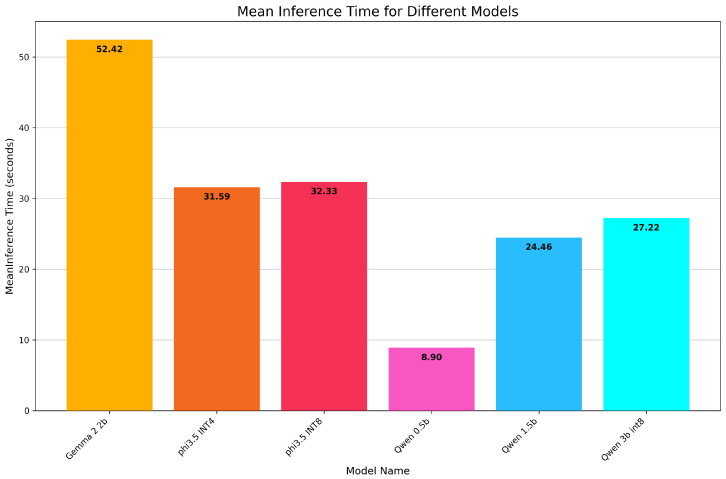
Average inference time for each SLM.

Figure 9 shows the average times for each SLM. Note that achieving low inference latency is not critical for our use case: sailors can typically tolerate response delays of within one minute, because, typically, a sailor approaching a port has a sufficient amount of time to interact with MarineChat.

Optimizations to minimize the inference time were mainly aimed at lowering the power consumption of the edge device, which is a crucial factor in maritime scenarios, where power resources are limited.

We can see that all models require reasonable amounts of time to generate a response. None takes more than a minute on average.

**Figure 10:**
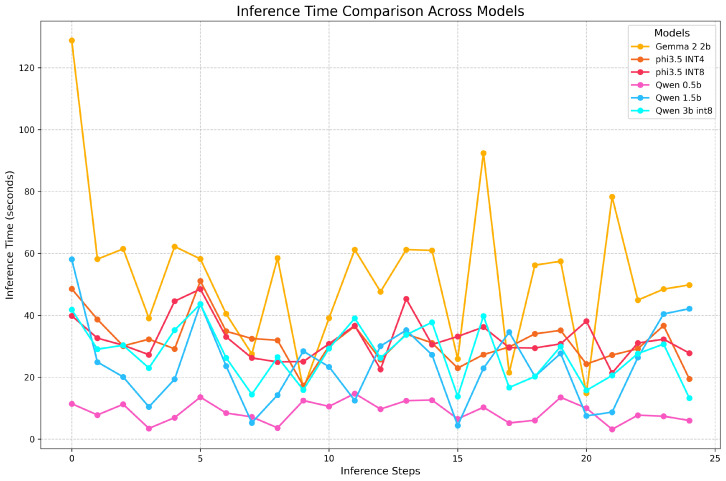
Comparison of the inference times for each question for the different SLMs.

If we analyze this aspect in detail, as shown in Figure 10, we can see that Gemma presents a considerably long inference time for the first question. This anomalous result could be due to the version of OpenVINO used in this work, i.e., 2024.3, which does not yet support the Gemma 2 models (2b and 9b). Moreover, the times for the subsequent questions are also slower than those of the other models, despite the fact that this is not the heaviest model.

Three models are very similar in terms of inference performance: Phi-3.5-mini INT8, Phi-3.5-mini INT4, and Qwen-2.5 3B INT8. Starting from comparable sizes (see Table 3), their quantization results in similar inference times. Notably, the smallest model, Qwen-2.5 3B INT8—which has only 3.09 billion parameters, in contrast to the largest model, Phi-3.5, with 3.82 billion—is also the fastest of the three.

Regarding these three models, it is interesting to note that, despite being quantized to a less precise 4-bit representation, the Phi-3.5-mini INT4 model has an inference time that is almost identical to that of its larger counterpart, Phi-3.5-mini INT8. This is because quantization was performed asymmetrically, with a ratio that retained 20% of the weights in INT8 precision. Consequently, at least in terms of inference performance (although the INT4 model is smaller in memory, taking up only 3 GB, compared to the 5 GB required for the INT8 version), there is essentially no difference between them.

Regarding the other two models, Qwen-2.5 1.5B shows intermediate performance compared to Qwen-2.5 0.5B and Qwen-2.5 3B INT8. Its inference times are slightly slower than those of Qwen-2.5 0.5B but faster than those of Qwen-2.5 3B INT8, making it a balanced option for those requiring moderate model capacity without the resource demands of larger models. Qwen-2.5 0.5B, on the other hand, is the fastest overall, with consistently low inference times. This is expected due to its much smaller size (0.5 billions parameters), making it an ideal choice for scenarios where the optimization of the power consumption of the edge device is paramount.

These results are summarized in Table 4. An in-depth analysis of the inference time and performance of each considered model can be found in Chapter 5 of [73].

#### Nvidia Jetson

We performed a subset of the experiments using an NVIDIA Jetson AGX Xavier board (NVIDIA), The Jetson AGX Xavier is a high-performance edge AI computing platform, designed primarily for robotics and autonomous machines. It is powered by an 8-core NVIDIA Carmel 64-bit ARMv8.2 CPU and a 512-core NVIDIA Volta GPU; equipped with 64 Tensor Cores, it includes 16 GB of 256-bit LPDDR4x memory and 32 GB of eMMC 5.1 storage It is capable of 32 TOPS at energy consumption between 10 and 30 W.

All parts of the pipeline that form MarineChat were maintained in the GPU, with the exception of the OpenVINO-related functions and classes. The model for the embedding step and the model for reranking were also the same, allowing for the clearest possible comparison of the results. Moreover, we reused the same index vector, and we asked the same queries.

**Figure 11:**
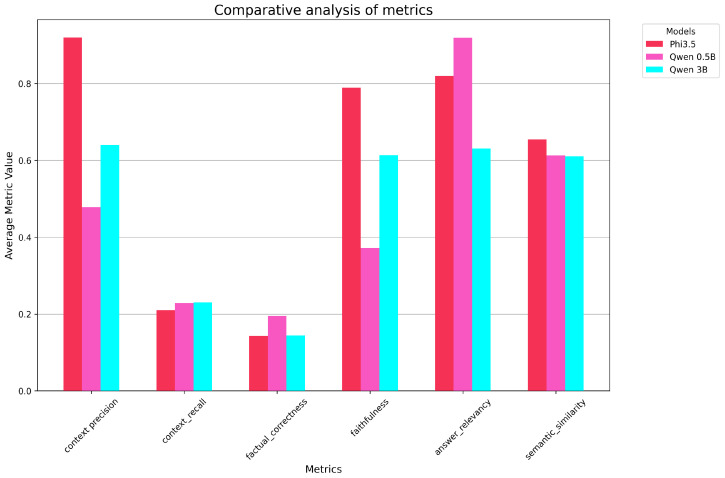
Means of the metrics when using ChatGPT-4o as the evaluator.

Figure 11 shows the metrics computed using the OpenAI models as evaluators. Unfortunately, we were not able to run the Gemma 2 and Qwen-2.5 1.5b models due to porting problems.

We note that the Phi-3.5-mini model performs the best in many metrics, such as context precision, which indicates that the retrieved context is used in the answer given by the model, or faithfulness, which again highlights the model’s ability to take into account the information found. The best results for this model on the GPU are achieved when using the model without quantization. Qwen-2.5 0.5b is the smallest model and yet it performs well overall. It even surpasses the larger models, especially in factual correctness and answer relevancy.

Regarding memory usage and inference times, these depend on the model and quantization, as shown in Table 4. The achieved results are very close to those obtained with the Xtreme CPU. Qwen-2.5 0.5b runs in 9.27 s, while the other two models require 28 s. We did not use any libraries, techniques, or frameworks specific to this GPU to speed up inference, because very low inference times are not necessary for our use case and because the focus was on the feasibility of the proposed MarineChat architecture on different edge devices.

Regarding the energy implications of using an edge device powered by a battery, a small yacht is typically equipped with 12 V 200 Ah batteries. This means that it is easily capable of powering these devices for more than 24 h. The expected consumption is much lower, considering that the maximum time per single response is less than a minute.

### 5.5. Upper Bound/Cloud Reference

Cloud-only tools are not comparable baselines under the stated constraints. However, for the sake of completeness, we compared the results with those obtained with an LLM service on the cloud. More specifically, we considered the use of a ready-to-use cloud service such as Google NotebookLM, where it is only necessary to upload the PDF of the pilot book, without any pre-processing step.

The results obtained for the prompts in Table 1 and Table 2 are as follows: faithfulness, 1.0; factual correctness, 1.0; context recall, 0.95; context precision, 1.0; answer relevancy, 1.0; and semantic similarity, 0.95. The good quality of the results is not surprising given the types of considered documents and prompts and the power of the Gemini model and the Google Cloud platform. However, it should be pointed out that the aim of our work was to demonstrate that reasonable answers can be obtained in environments with limited internet access, which often prevent the use of cloud services and LLMs such as Google NotebookLM and ChatGPT.

## 6. Conclusions and Future Developments

In this work, we have presented a quantitative and qualitative evaluation of the effectiveness of systems based on language models on edge devices to support users in making decisions, as in the considered maritime use case. Particular attention has been paid to tackling the expected limitations due to the reduced computing capabilities of such devices.

First, we considered the use of SLMs, which are, in principle, able to operate also on edge devices. We considered also the RAG technique to improve the quality of the generated responses, given the particular context of our use case, where SLMs struggle due to their intrinsic limitations. The result is represented by MarineChat, our prototype RAG chat-bot designed to be used by sailors on small/medium-sized boats to obtain port information for their needs, even if an internet connection is not available.

From a qualitative point of view, all tested models are able to remain faithful to the user’s questions and to the information retrieved to answer them in the majority of cases, while the inference times are below one minute—a response delay that can be well tolerated by sailors approaching a port. We performed the tests on a limited set of 25 questions and considering only the Italian coastline subset, in line with the expertise of some of the authors. Nevertheless, on the basis of the architecture of the system, we expect similar behavior for all ports if the corresponding pilot book is included in the RAG component.

On the other hand, the results show that the context retrieved by the RAG pipeline is often not useful or inserts irrelevant information, which can affect the correctness of the model answers. This issue is reflected in the generally low values of the context recall metric. Moreover, this aspect is reflected in the factual correctness metric, which indicates whether the generated response aligns with the provided reference and generally shows very low values.

Having proven the effectiveness of MarineChat, we will focus our future research activities on the following.

- First, we plan to improve the effectiveness of the RAG pipeline (1) by improving the clarity of the dataset using post-processing techniques such as extracting information from images and tables. (2) by searching for the best parameter configurations (e.g., the chunk size and the threshold score); and (3) by testing different embeddings [74] and reranking. models. In particular, we will experiment with the use of non-quantized rerankers; so far, we have used quantization to reduce the execution times, but, given the achieved results, we can trade off some speed for improved performance. To strengthen our results, we will also consider more questions and several coastline portions in different countries.
- The second improvement will be the integration of real-time data, which, on ships, is generated by a number of heterogeneous sensors [75], in order to further improve the effectiveness of the responses.
- Another possible direction for future development is the integration of textual responses with nautical charts restricted to the relevant area. While pilot books and nautical charts have traditionally been considered separate tools, indispensable in providing integrated information for safe navigation, merging them into a single digital tool offers significant added value. However, it remains to be analyzed whether the benefits balance out the cost in terms of necessary resources.
  A simple solution aiming to minimize additional resource consumption could be tool-augmented generation, based on a post-processing procedure on the text response.
  A more advanced solution could employ multimodal models, albeit at the cost of increased resource consumption and the risk of hallucination. These SLM-based models have the ability to produce multimodal information in various formats, such as images, audio, and video [32,76,77,78]. For the same reason, other RAG methods will be considered, such as hybrid RAG [79], adaptive retrieval like Self-RAG [80], or RAG frameworks designed to exploit diverse static data formats, such as images [81] or maps.
- Lastly, MarineChat will be provided with a vocal interface and integrated into onboard boat devices, such as modern chart plotters, in order to render it more user-friendly for real-world applications.

## Figures and Tables

**Table 1:** Questions and ground truths: part I.

Question	Ground Truths
What are the typical winds in Genoa?	The typical winds in Genoa are from the north (Tramontana) to the north-west in winter, and from the south-east (Scirocco) and the south-west (Libeccio) in summer.
What is the depth of the sea in the Port of Alassio?	The average depth in the Port of Alassio is 5 m.
What are the VHF channels of the Port of Imperia?	There are two VHF channels in the Port of Imperia: 12 and 16.
Where is the Golfo Marconi situated?	Marconi Gulf is located between Portofino and Chiavari, along the eastern Ligurian Riviera.
Could you please provide me with the email address for the Port of Ischia?	Ischia Harbor Email is ischia@guardiacostiera.it.
Could you explain how to enter the Port of Genoa? What instructions should I follow, and what should I watch out for?	To enter the Port of Genoa, one must have a moderate speed and be careful to use the right entrances for pleasure boats and control the movement of larger ships of all types as it is a very large port. If possible, contact the port offices first.
What is the intensity of the tides at Ancona Port?	Tides in the Port of Ancona are generally negligible, only reaching 0.5 m and up to a maximam of 0.9 m when the strong N wind (bora) blows.
What are the main dangers in the Port of Naples?	The main dangers in the Port of Naples are the many fast-sailing tourist ferries.
Which winds are sheltered from in the harbor of Otranto?	To all except N winds, which can be very violent, especially in winter.
Can you land on Pianosa Island in the Tremiti Islands?	No, because it is a marine protected area. One must keep a distance of 500 m from its coastline.
What are the most prominent landmarks for identifying Capo Mele from the sea?	A light is shown from a prominent tower with a dwelling, 25 m high, standing on the cape. A signal station surmounts the summit of the cape.
Where can I anchor near the Port of San Remo?	Vessels can anchor at a depth of 12 m, east of the harbor entrance. The holding ground is good, but anchorage is not advisable with strong south (S) winds.
Can you describe the Port of Carloforte?	Carloforte has a small harbor protected by two breakwaters, one almost perpendicular to the other, forming a narrow entrance.

**Table 2:** Questions and ground truths: part II.

Question	Ground Truths
Is there a VHF channel that can provide information about passing through the Strait of Messina?	The VHF channels are 10, 13, 14, and 16.
Which winds does the Port of Trapani face?	The harbor is exposed to all winds and is open to the W. In the spring, strong seasonal SE winds can make entry hazardous.
What is the Marrobbio phenomenon?	Marrobbio is a rare meteorological phenomenon observed in the Mediterranean, particularly along the southwestern coast of Sicily. It involves sudden and significant oscillations in sea level, similar to a small tsunami, caused by atmospheric pressure changes and wind patterns. These rapid changes can lead to temporary flooding in coastal areas.
What is the largest vessel that can be accommodated at San Remo Port?	Vessels up to 100 m in length and with a draft of 4.3 m can be accommodated at the Port of San Remo.
What navigational hazards are there near the island of Giglio in the Tuscan Archipelago?	A restricted area lies 4 miles W of Isola Giglio. Mariners should consult local authorities before entering the area.
What dangers might there be when entering the Port of Civitavecchia at night?	Care should be exercised when entering the harbor at night as the navigation lights are not easily distinguished from the lights of the town.
Could you describe the typical winter winds that affect the Port of Trieste?	From September to March, winds alternate usually between the NW and NE. The NE winds sometimes blow with such force as to cause a sea in Mare Piccolo. Winds from the SW raise a lively sea and cause high water in Mare Grande. Winds from the SE, especially in winter, are strong and may disturb cargo handling by vessels berthed in Porto Mercantile.
What are the currents like in the Port of Chioggia?	Currents are strong and are mostly influenced by rain, runoff, and winds. With strong winds from the NE and SE, the current between the breakwaters can attain a rate up to 4 knots.
What is the draught depth at Rimini Port?	The harbor has depths of 2.9 to 3.7m and is subject to silting.
Is Portoferraio Bay safe for anchoring?	The roadstead of Portoferraio affords anchorage to vessels of all sizes, but the bottom, being of soft mud, is not a good holding ground. With SE winds, which prevail in the area, vessels are liable to drag anchor, even when riding to two anchors. It is important to stay clear of the ferry turning areas and the harbor approaches. While ferry wash can sometimes cause some movement, it does not pose a significant danger for anchored vessels.

**Table 3:** The different models tested, with their size in terms of the number of parameters, the size in gigabytes (GB) before (BQ) and after quantization (AQ), and the type of quantization. The letter B stands for billions, and Size (B) indicates the size per billion parameters.

Vendor/Name	Size (B)	Size (GB) BQ	Size (GB) AQ	Quantization
Phi-3.5-mini-instruct	3.82	7.11	2.28/3.56	INT4 ASYM/INT8
Qwen-2.5-0.5B-Instruct	0.494	0.953	0.953	None
Qwen-2.5-1.5B-Instruct	1.54	2.88	2.88	None
Qwen-2.5-3B-Instruct	3.09	5.75	2.92	INT8
Gemma-2-2b-it	2.61	4.88	4.88	None

**Table 4:** On-device efficiency. Xtreme: Intel UP Xtreme i14 (CPU Core Ultra 7); Xavier: NVIDIA Jetson AGX Xavier (GPU Volta); P: number of parameters; Q: quantization; MS: model size; RAM: maximum RAM size; AIT: average inference time.

Device	Model	P	Q	MS (GB)	RAM (GB)	AIT (s)
Xtreme	Qwen-2.5-0.5B-Instruct	0.494B	None	0.953	1.2	8.90
Xtreme	Qwen-2.5-1.5B-Instruct	1.54B	None	2.88	3.5	24.46
Xtreme	Qwen-2.5-3B-Instruct	3.09B	INT8	5.75	7	27.22
Xtreme	Phi-3.5-mini-instruct	3.82B	INT4	2.28	3	31.59
Xtreme	Phi-3.5-mini-instruct	3.82B	INT8	3.56	4.5	32.33
Xtreme	Gemma-2-2b-it/None	2.61B	None	4.88	6	52.42
Xavier	Qwen-2.5-0.5B-Instruct	0.494B	None	0.953	1.5	9.27
Xavier	Qwen-2.5-3B-Instruct	3.82B	None	5.75	7.4	28.43
Xavier	Phi-3.5-mini-instruct	3.82B	None	7.11	7.9	28.98

## Data Availability

The data presented in this study are openly available in World Port Index https://msi.nga.mil/Publications/WPI (accessed on 10 July 2024).

## Abbreviations

    The following abbreviations are used in this manuscript:

AI	Artificial Intelligence
LLM	Large Language Model
NLP	Natural Language Processing
RAG	Retrieval-Augmented Generation
SLM	Small Language Model
